# Effects of personalized diets by prediction of glycemic responses on glycemic control and metabolic health in newly diagnosed T2DM: a randomized dietary intervention pilot trial

**DOI:** 10.1186/s12916-022-02254-y

**Published:** 2022-02-09

**Authors:** Michal Rein, Orly Ben-Yacov, Anastasia Godneva, Smadar Shilo, Niv Zmora, Dmitry Kolobkov, Noa Cohen-Dolev, Bat-Chen Wolf, Noa Kosower, Maya Lotan-Pompan, Adina Weinberger, Zamir Halpern, Shira Zelber-Sagi, Eran Elinav, Eran Segal

**Affiliations:** 1 Department of Computer Science and Applied Mathematics, Weizmann Institute of Science, 7610001 Rehovot, Israel; 2 Department of Molecular Cell Biology, Weizmann Institute of Science, 7610001 Rehovot, Israel; 3 School of Public Health, University of Haifa, 3498838 Haifa, Israel; 4 Pediatric Diabetes Unit, Ruth Rappaport Children’s Hospital, Rambam Healthcare Campus, Haifa, Israel; 5 Immunology Department, Weizmann Institute of Science, 7610001 Rehovot, Israel; 6 Digestive Center, Tel Aviv Sourasky Medical Center, 6423906 Tel Aviv, Israel; 7 Internal Medicine Department, Tel Aviv Sourasky Medical Center, 6423906 Tel Aviv, Israel

**Keywords:** Type 2 diabetes mellitus, Gut microbiome, Dietary intervention, Postprandial glucose responses, Personalized nutrition

## Abstract

**Background:**

Dietary modifications are crucial for managing newly diagnosed type 2 diabetes mellitus (T2DM) and preventing its health complications, but many patients fail to achieve clinical goals with diet alone. We sought to evaluate the clinical effects of a personalized postprandial-targeting (PPT) diet on glycemic control and metabolic health in individuals with newly diagnosed T2DM as compared to the commonly recommended Mediterranean-style (MED) diet.

**Methods:**

We enrolled 23 adults with newly diagnosed T2DM (aged 53.5 ± 8.9 years, 48% males) for a randomized crossover trial of two 2-week-long dietary interventions. Participants were blinded to their assignment to one of the two sequence groups: either PPT-MED or MED-PPT diets. The PPT diet relies on a machine learning algorithm that integrates clinical and microbiome features to predict personal postprandial glucose responses (PPGR). We further evaluated the long-term effects of PPT diet on glycemic control and metabolic health by an additional 6-month PPT intervention (*n* = 16). Participants were connected to continuous glucose monitoring (CGM) throughout the study and self-recorded dietary intake using a smartphone application.

**Results:**

In the crossover intervention, the PPT diet lead to significant lower levels of CGM-based measures as compared to the MED diet, including average PPGR (mean difference between diets, − 19.8 ± 16.3 mg/dl × h, *p* < 0.001), mean glucose (mean difference between diets, − 7.8 ± 5.5 mg/dl, *p* < 0.001), and daily time of glucose levels > 140 mg/dl (mean difference between diets, − 2.42 ± 1.7 h/day, *p* < 0.001). Blood fructosamine also decreased significantly more during PPT compared to MED intervention (mean change difference between diets, − 16.4 ± 37 μmol/dl, *p* < 0.0001). At the end of 6 months, the PPT intervention leads to significant improvements in multiple metabolic health parameters, among them HbA1c (mean ± SD, − 0.39 ± 0.48%, *p* < 0.001), fasting glucose (− 16.4 ± 24.2 mg/dl, *p* = 0.02) and triglycerides (− 49 ± 46 mg/dl, *p* < 0.001). Importantly, 61% of the participants exhibited diabetes remission, as measured by HbA1c < 6.5%. Finally, some clinical improvements were significantly associated with gut microbiome changes per person.

**Conclusion:**

In this crossover trial in subjects with newly diagnosed T2DM, a PPT diet improved CGM-based glycemic measures significantly more than a Mediterranean-style MED diet. Additional 6-month PPT intervention further improved glycemic control and metabolic health parameters, supporting the clinical efficacy of this approach.

**Trial registration:**

ClinicalTrials.gov number, NCT01892956

**Supplementary Information:**

The online version contains supplementary material available at 10.1186/s12916-022-02254-y.

## Background

Type 2 diabetes mellitus (T2DM) is a progressive disease characterized by increased blood glucose levels that lead to serious macro and microvascular complications [1, 2]. The prevalence of T2DM is increasing worldwide, affecting ~ 10% of the global population [3, 4]. Thus, seeking for effective prevention and treatment solutions for T2DM is of high priority.

The primary goal in T2DM management is to improve glycemic control to reduce the risk for health complications. Blood glycated hemoglobin (HbA1c), which is a marker for a 3-month average of blood glucose levels, is often used to assess glycemic control in diabetes management [5]. Recently, continuous glucose monitoring (CGM) devices have become appreciated as another reliable tool for evaluating glycemic control in research settings and clinical practice [6].

Dietary modifications prominently affect glycemic control and have a fundamental role in T2DM management. Moreover, in newly diagnosed T2DM, they may delay the introduction of diabetes medications or even result in diabetes remission [7, 8]. Particularly, postprandial glucose responses (PPGR) are considered a major determinant of glycemic control and are usually targeted in clinical practice by the meal “carbohydrate counting” approach [9, 10], although it is insufficiently predictive of PPGRs [11]. Indeed, there is no consensus on the ideal amount of dietary carbohydrates in T2DM management, and low-carbohydrate diets are not proven superior to high-carbohydrate diets in their capacity to impact long-term glycemic control [12, 13]. Other methods aimed at estimating PPGRs are the glycemic index and the derived glycemic load [14]. However, these methods quantify PPGRs to consumption of single tested foods or meals and thus have limited applicability in assessing PPGRs to real-life meals [15]. Studies examining the effect of low glycemic index diets on T2DM risk, weight loss, and cardiovascular risk factors yielded mixed results [16–18]. Other strategies for improving glycemic control in T2DM include eating patterns such as the Mediterranean-style diet, which has beneficial effects on glycemic control and metabolic health [19, 20]. However, in clinical practice, many patients fail to achieve clinical goals with diet alone, suggesting that alternative and personalized dietary strategies are needed to achieve glycemic control.

We previously reported high interpersonal variability in PPGRs to identical meals in an 800-person cohort and developed a machine learning algorithm for predicting personalized PPGRs to any given meal, using clinical and gut microbiome features [21].

Here, we sought to evaluate whether dietary interventions based on our algorithm improve PPGRs in individuals with newly diagnosed T2DM and naive to glucose-lowering medications, as compared to a commonly recommended Mediterranean-style diet. We further sought to evaluate the long-term clinical effects of the algorithm-based diet on glycemic control and metabolic health, to address its applicability in clinical practice as well as its effects on gut microbiome composition.

## Methods

### Study design

This study was a single-center randomized crossover dietary intervention followed by an additional 6-month single-arm intervention performed at the Weizmann Institute of Science, Israel. The trial was conducted between October 2017 and October 2019. No changes were done to the study protocol and methods after the trial commenced. The trial was a pilot study for a “proof-of-concept” of the clinical efficacy of an algorithm-based diet in T2DM, and as such it was included as part of a former approved protocol of another trial (“Personalized Nutrition Project,” hereafter “PNP” [21]). The PNP protocol was approved by Tel Aviv Sourasky Medical Center Institutional Review Board (IRB), approval number TLV-0658-12. All participants enrolled for the present trial provided written informed consent.

### Study population

**Fig. 1:**
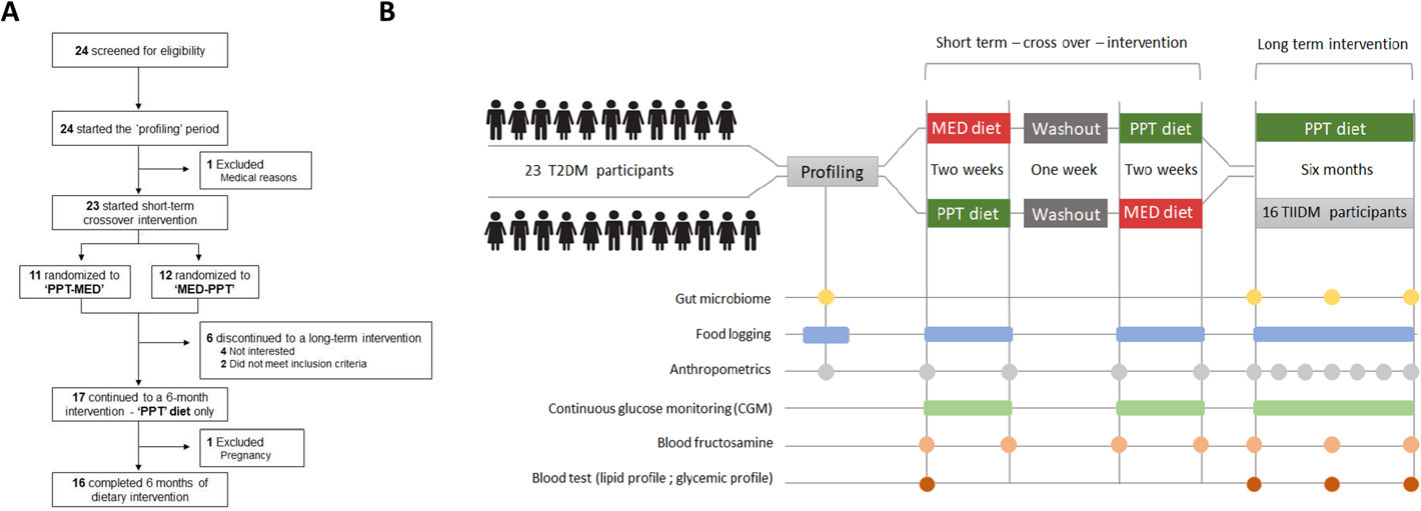
Trial flow and study outline. **A** Diagram of trial flow. **B** Illustration of the experimental design, comparing the effects of following a 2-week long MED diet vs. a PPT diet on glucose levels and the effect of an additional 6-month PPT intervention program on multiple metabolic parameters

Twenty-three subjects with newly diagnosed type 2 diabetes were included in the short-term crossover intervention. Sixteen out of the 23 participants were included in the long-term (6 months) PPT intervention program. Recruitment, randomization, and flowchart numbers are detailed in Fig. 1A. Screening for the trial was done at the trial’s central lab as part of a screening process for a larger-scale study in subjects with prediabetes [22], and participants were directed to the present trial if the lab test results met the glycemic values as specified in the inclusion criteria of this trial. In total, 24 subjects met the following inclusion criteria: age, 18–65; HbA1c, 6.5–8% or fasting plasma glucose (FPG), 140–180 mg/dl; naive to glucose-lowering medications; and capability to work with a smartphone application on a daily basis (for food logging). The key exclusion criteria included a recent treatment (last 3 months) with antibiotics/antifungal, use of anti-diabetic and/or weight-loss medication. Urine tests were also performed at screening to detect protein levels and eliminate cases of proteinuria which may indicate a progressive stage of diabetes with microvascular complications (for a full list, see Additional file 1, Supplemental Methods, page 3). One participant was excluded before the beginning of the intervention (day 0), due to the high levels of HbA1c detected at that time point, resulting in a total of 23 participants that started and completed the crossover intervention. Prior to the intervention, participants went through a profiling stage, including medical and dietary history obtained from questionnaires and meeting with a dietitian, stool sample for microbiome profiling (required for algorithm predictions), anthropometric measurements, and 3-day food logging. Upon completion of the crossover intervention, data were analyzed and results were revealed to participants who were offered to proceed to a long-term single-arm intervention with the personalized postprandial targeting (PPT) diet only. No significant differences were found at the end of the crossover intervention between participants who proceeded vs. did not proceed to the long-term intervention (Additional file 1: Table S1). No relevant diet-related adverse effects were observed. Blood tests were reviewed by a physician from the trial team, and participants were notified in case of abnormal results that require further medical assessment. At the end of each intervention (short-term crossover and long-term 6-month intervention), participants received a summary report with all their personal measurements tested during the intervention.

### Intervention

The study outline and the collection of various measurements throughout the trial are detailed in Fig. 1B. After completing the profiling stage, participants were randomized by one programmer from the trial personnel, who had no contact with the participants, to one of the two allocations with no restrictions and started the crossover dietary intervention in a sequence according to the study arm they were randomized to (“PPT-MED” or “MED-PPT”), see Table 1 for the baseline characteristics of the two groups.

**Table 1:** Baseline characteristics of participants

Parameters (units)	All (***n*** = 23)	PPT-MED (***n*** = 11)	MED-PPT (***n*** = 12)	***p*** value*
Age (years)	53.5 ± 8.9	55.6 ± 7.8	51.5 ± 9.8	0.27
Male sex, number (%)	11 (48)	5 (45)	6 (50)	0.84
**Anthropometric measurements**				
Weight (kg)	87.5 ± 21.5	90.1 ± 22.9	85.1 ± 20.8	0.59
BMI (kg/m^2^)	30.8 ± 7.4	33.0 ± 8.9	28.8 ± 5.3	0.18
Waist circumference (cm)	102 ± 14.8	105 ± 15.4	99.4 ± 14.3	0.4
Body fat (%)	33.7 ± 10.9	36.3 ± 12.2	31.5 ± 9.8	0.33
**Lipid profile**				
Serum total cholesterol (mg/dl)	200.7 ± 87.2	190.7 ± 29.6	211.0 ± 50.1	0.35
Serum LDL cholesterol (mg/dl)	125.6 ± 56.9	121.9 ± 22.5	127.8 ± 83.8	0.71
Serum HDL cholesterol (mg/dl)	47.4 ± 20.1	44.4 ± 7.7	49.2 ± 9.4	0.27
Serum triglycerides (mg/dl)	153.5 ± 76.4	151.9 ± 57	154.4.9 ± 53.1	0.92
**Glucose biomarkers**				
HbA1c (%)	6.8 ± 0.4	6.8 ± 0.3	6.8 ± 0.5	0.73
Fructosamine (μmol/l)	295.1 ± 35.5	292.8 ± 44.1	297.2 ± 27.3	0.78
Fasting plasma glucose (mg/dl)	149.7 ± 19	153 ± 17.8	146.7 ± 20.4	0.44

*Abbreviations*: *BMI* body mass index, *PPT* personalized postprandial-targeting diet, *MED* Mediterranean diet

*p values for the differences between the groups were calculated using the *t*-test

Menus for both diets were constructed at this point according to the dietary guidelines of each dietary approach while including participants’ food preferences and caloric target which was determined to support estimated energy needs and with no intent for caloric restriction and was used to construct menus for participants for the crossover and the long-term interventions (Fig. S1A, supplementary methods [23]). Notably, during the crossover intervention, participants received a fixed menu consisting of 4–5 options of meals for breakfast, lunch, and dinner and 10–12 options of meals for snack. Participants self-prepared their assigned meals at home. They were instructed to consume the exact foods as appeared on their assigned menus, while using a digital kitchen scale provided to them during the study to assure the accuracy of food amounts consumed.

#### Mediterranean (MED) diet

The dietary recommendations for the Mediterranean dietary approach were based on the Mediterranean diet (hereafter “MED diet”) guidelines which are commonly recommended for T2DM patients in order to improve glycemic control and reduce the risk of metabolic complications [24, 25] and were defined by 4 outside dietitians. Recommended foods on the MED diet included whole-wheat bread and grains, legumes, low-fat dairy products, fish, poultry, olive oil, fruits, and vegetables. Discouraged foods included commercial bakery goods, sweets and pastries, fried foods and snacks, fatty and processed meat, and high-fat dairy products. Menus in the MED diet were designed with the following diet composition: 45–65% of energy intake from carbohydrates, 15–20% from protein, and < 35% from fat, with < 10% from saturated fat.

#### Personalized postprandial targeting (PPT) diet

The dietary recommendations in the personalized postprandial-targeting diet (hereafter “PPT diet”) intervention were personally tailored to participants based on predictions of their glycemic responses, according to an algorithm that originated from a previous work in our lab (see Additional file 1, Supplemental Methods, page 4) [21]. The algorithm used in the present study was adjusted for use in a clinical setting according to the study design and integrated personal data of blood test results (HbA1c, FPG, and hemoglobin), microbiome features (abundances), anthropometric features (such as weight and waist circumference), health questionnaires, and dietary components of the meals (see Additional file 2 for the full list of features). Notably, CGM-based features were not used for prediction in the present trial, as there was no glycemic profiling with CGM prior to the intervention. As opposed to the MED diet, the PPT diet did not rely on the predetermined distribution of macronutrients or any uniform set of recommendations. The selection of meals to menus in this dietary intervention relied on a scoring system that we developed (see Fig. S1B). During the long-term period, participants received menus with multiple (hundreds) choices of meals and snacks and were provided with an interactive feature on their smartphone logging app, which provided them real-time feedback on any other desired food or meal outside of their menus, whether it is personally recommended for them or not, based on the algorithm predictions (Fig. S1C).

During each of the dietary intervention periods (both short term and long term), participants were connected to CGM sensors (*Freestyle Libre*, Abbott) with sensors replaced every 2two weeks (mean ± SD of 16,600 ± 3300 glucose measurements per participant) and were blinded to glucose tracings. Furthermore, participants were invited to follow-up meetings once a month at the study site and could contact their personal dietitian in between.

### Adherence to the study

The participants’ adherence to the prescribed diets during the crossover intervention was evaluated by the dietitian, through close monitoring of their self-recorded dietary intake in the logging application throughout the 2 weeks of each dietary intervention. In the long-term 6-month PPT intervention, adherence was also assessed based on the self-recorded dietary intake in the logging app, as well as by monthly electronic follow-up questionnaires that participants were asked to fill out. In order to encourage dietary adherence and self-monitoring, we generated semi-automatic feedback reports for participants every 2 weeks. These feedback reports included composite grades on a scale of 0–100 (from worse to best) for diet composition and calorie intake separately for the entire 2-week period as detailed below and an additional list of “best” and “worst” logged meals.

- PPT diet composition grade indicates how well the participant sticks to predictor-based meal scores. Each meal score was assigned with a grade as follows: meal score 1 = grade 100, meal score 2 = 80, meal score 3 = 50, meal score 4 = 25, and meal score 5 = 0. The grades are averaged calorie-wise (with food energy trimmed to be within (100,500) kcal interval)- Σ kcal(*i*) grade(*i*). For example, if a person ate three meals: 600 kcal of meal score 2, 1000 kcal of meal score 5, and 80 kcal of meal score 1, feedback grade would be: (500 × 80 + 500 × 0 + 100 × 100)/(500 + 500 + 100) = 45. If too few (100 by default), calories are logged (overall), we did not compute a score.
- Calories grade indicates how well the participant sticks to the prescribed caloric target. When caloric intake deviates within 15% of caloric target (CT), the applied grade is 100; when caloric intake deviation exceeds, 60% of CT the applied grade is 0; when caloric intake deviation is between 15 and 60%, a linear penalty is applied to the grade depending on the deviation.

### Outcome variables

Outcome measures in the short-term crossover intervention were taken at the beginning and end of each diet. In the long-term intervention, clinical outcomes obtained from blood tests and stool samples were measured at the beginning (hereafter “T0”), middle (hereafter “T3”), and end (hereafter “T6”) of intervention. Body composition measurements were measured monthly (Fig. 1B). The primary outcome measures for the crossover intervention included (1) blood fructosamine levels and (2) meal PPGR (quantified as the incremental area under the glucose curve (iAUC) in the 2 h after the meal was logged). Secondary outcomes included body composition measurements (using a Segmental Body Composition Analyzer; BC-418MA Tanita, Tokyo, Japan) and CGM-based glycemic measures (including daily duration (in hours) of glucose levels above 140, 150, 160, 170, 180 mg/dl; glucose fluctuations measured by the ratio between the standard deviation and mean of blood glucose levels; and averaged PPGR, calculated as the iAUC of every 9 glucose measurements during every connection). The primary outcome for the long-term intervention included the change in HbA1c levels. The secondary outcomes included (1) blood tests for FPG, fructosamine, fasting insulin, homeostatic model assessment for insulin resistance (HOMA-IR), lipid profile (total cholesterol, low-density lipoprotein (LDL) cholesterol, high-density lipoprotein (HDL) cholesterol, triglycerides), liver enzymes (alanine aminotransferase (ALT), aspartate aminotransferase (AST), and gamma-glutamyl transferase (GGT), kidney function (creatinine, urea), and C-reactive protein (CRP); (2) CGM-based glycemic measures as in the short-term intervention; (3) body composition measures as in the short-term intervention and also waist and hips circumferences taken by the participant’s dietitian at baseline, T0 and T6; (4) blood pressure measurements, taken with an automated blood pressure monitor (M6 model, Omron, Hoofddorp, the Netherlands) at baseline, T0, and T6; and (5) microbiome composition and function based on stool samples collected at baseline, T0, and T6.

### Laboratory testing

Blood draws were done at the trial site (Weizmann Institute of Science) or at the central medical laboratory of the trial (AMC Medical Center Laboratory, LTD). All blood specimens were processed and lab tests performed by one lab technician at the central laboratory, who was unaware of the arm assignment or any other characteristics of participants. HbA1c determination was based on the turbidimetric inhibition immunoassay (TINIA) for hemolyzed whole blood, standardized according to IFCC transferable to DCCT/NGSP (Tina-quant HbA1c Gen. 3 assay, cobas, Roche) [26]. Plasma glucose was measured with the use of a hexokinase method (GLUC2 assay, cobas, Roche). Fructosamine was measured with the use of a colorimetric test by reaction with nitroblue tetrazolium (FRA assay, cobas, Roche) [27]. Insulin was measured with a one-step immunoassay to determine the presence of human insulin in human serum or plasma, using CMIA technology (ARCHITECT Insulin assay, Abbot). For lipid profile, cholesterol was measured with the use of enzymatic, colorimetric method (CHOL2 test, cobas, Roche), HDL cholesterol was measured with homogeneous enzymatic colorimetric test (HDLC4, cobas, Roche) [28]. Triglyceride level was measured with enzymatic colorimetric test (TRIGL assay, cobas, Roche/Hitachi).

### Gut microbiome sampling and sequencing

Subjects provided stool samples that were self-sampled using the OMNIgeneGUT (OMR-200; DNA Genotek) stool collection kit. For shotgun sequencing, DNA was purified using the PowerMag Soil DNA isolation kit (MoBio) optimized for the Tecan automated platform. DNA was diluted to 1.5 ng, and Illumina libraries were prepared using Nextera DNA library preparation kit, Ref# 15028211; by Tecan Freedom Evo 200 robot device. Nextera DNA Unique Dual Indexes Sets A–D from IDT were used for library preparation. Library concentration was measured using the iQuantTM dsDNA HS Assay Kit, ABP biosciences (Cat# AP-N011) and library size quantified by automated electrophoresis nucleic acid QC -Tape-Station system. Libraries were sequenced to a minimum depth of 10 million reads by NextSeq 500 device with IlluminaNS 500/550 High Output V2 75 cycle kit, Cat# FC-404-2005. Length-normalized RA of genes were assigned and obtained by similar mapping with GEM to the reference catalog of Li and colleagues [29].

### Gut microbiome analyses

The host DNA was detected by mapping with GEM [30] to the human genome with inclusive parameters, and these reads were removed. Bacterial relative abundance (RA) estimation was performed by mapping bacterial reads to species-level genome bins (SGB) representative genomes 10 K report meeting. We selected all SGB representatives with at least 5 genomes in a group, and for these representative genomes, kept only unique regions as a reference data set. Mapping was performed using bowti e[31], and abundance was estimated by calculating the mean coverage of unique genomic regions across the 50% most densely covered areas as previously described [32].

### Statistical analysis

Differences in dietary intake, daily time with glucose levels above the thresholds, and average PPGR between PPT diet and MED interventions were evaluated using the two-sided *t*-test, with *p*-values < 0.05 considered significant. The analysis of the mean change difference of fructosamine between treatments in the crossover trial was done using linear mixed models, using “statsmodels” library v.0.10.1 of Python 3.5. The within-participant differences in the average meal PPGR between diets during the crossover intervention were estimated using the Mann-Whitney non-parametric test. Differences in clinical outcomes and gut microbiome (relative abundances (RA)) during the long-term intervention were evaluated using the one-sample *t*-test. For the analysis of the association between gut microbiome changed, and clinical outcomes we used Pearson correlation. In the gut microbiome analysis, the results were corrected for multiple hypotheses testing using a false discovery rate (FDR) of 0.15 for each phylogenetic level separately.

## Results

### PPGR prediction in T2DM subjects

**Fig. 2:**
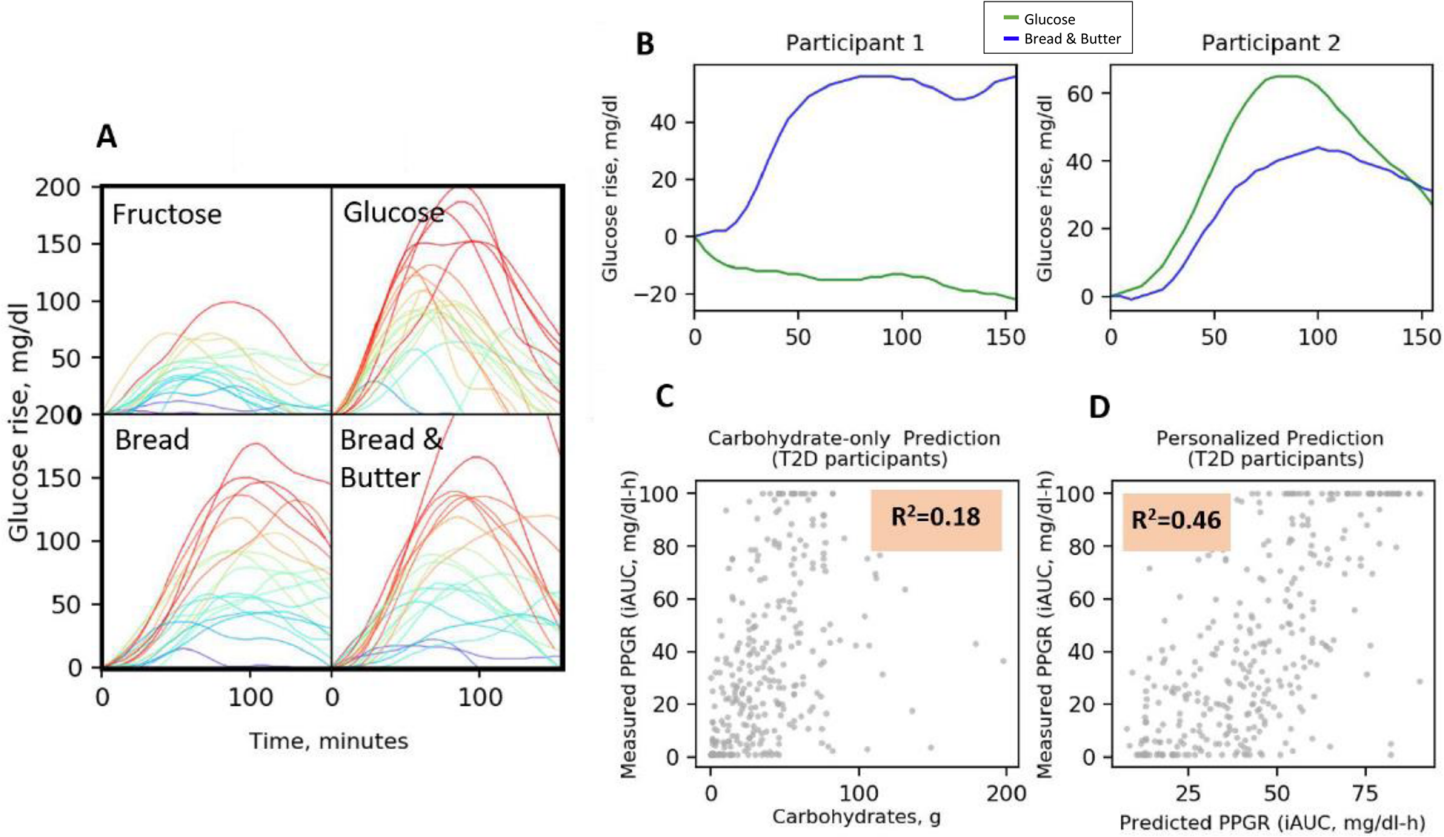
High interpersonal variability in the postprandial glucose responses of subjects with T2DM. Patterns and predictions of postprandial glucose responses (PPGR) in a subset cohort of subjects with newly diagnosed T2DM from a previous study [21]. **A** Glucose response after consuming standardized meals (bread, bread and butter, glucose, and fructose, each consisting of 50 g of available carbohydrates). Each line represents a different participant; participants are colored according to the level of glucose as measured by the CGM. Range of PPGRs from 0 to 100 mg/dl × h. **B** Example of the PPGR to two standardized meals for two participants exhibiting opposite PPGRs. Each meal contains 50 g of carbohydrates. **C** PPGR predictions across 22 newly diagnosed T2DM participants. Dots represent predicted (*x*-axis) and CGM-measured PPGR (*y*-axis) for meals, based only on the meal’s carbohydrate content. **D** The same as **C**, but here, the model was based on our predictor evaluated in leave-one person-out cross-validation on 22 newly diagnosed T2DM participants

To evaluate the applicability of our previously published machine-learning algorithm for PPGR prediction to subjects with T2DM, we analyzed the PPGRs of a subset of subjects from a previous cohort [21], who had HbA1c > 6.5%. Similar to our findings from previous studies on high interpersonal variability in PPGRs to meals among healthy [21] and prediabetes [22] individuals, we found high interpersonal variability in PPGRs also among subjects with T2DM (Fig. 2A, B). Applying our algorithm for PPGR prediction by clinical and gut microbiome features to this subset of T2DM subjects, we found that even in this population, the standard “carbohydrate counting” approach poorly explains the variability in PPGRs (18%), while adding clinical and microbiome features used by our algorithm increases the explained variance substantially (46%) (Fig. 2C, D).

### Crossover intervention

**Fig. 3:**
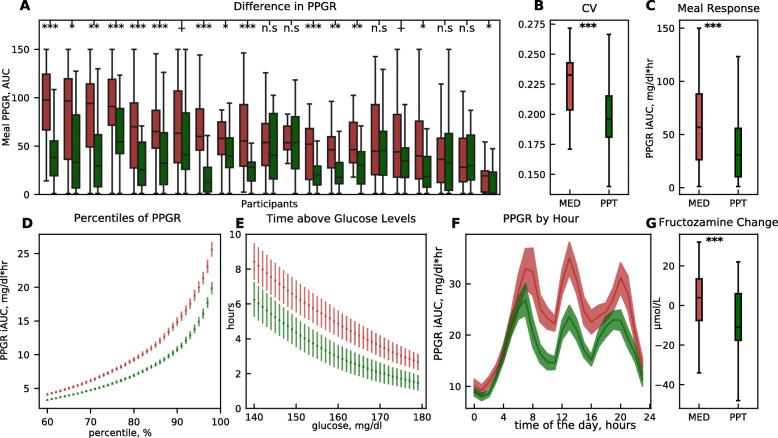
A PPT diet improves glycemic outcomes compared to the MED diet. Comparison of CGM-based glucose measures and fructosamine in the PPT diet (green) vs. MED diet (red), across all participants. **A** Boxplot of meal PPGRs during the MED diet (red) and PPT diet (green) interventions for all participants. Statistical significance is marked (Mann-Whitney *U* test ***p < 0.001, **p < 0.01, *p < 0.05, ^+^*p* < 0.1; n.s, not significant). **B** As in **A** but for blood glucose fluctuations (coefficient of variation) across all participants during each of the diets. Defined as the ratio between the standard deviation and the mean of blood glucose levels during each of the diets (LMM, *p* < 0.001). **C** As in **A** but for the average meal PPGR across all participants during each of the diets (LMM, *p* < 0.001). **D** Percentiles of PPGRs from continuous glucose measurements across all participants throughout the MED diet (red) and the PPT diet (green) interventions. **E** Number of daily hours (*y*-axis) above glucose level thresholds (*x*-axis), across all participants throughout the MED diet (red) and PPT diet (green) interventions. **F** Average PPGR (*y*-axis) during hours of the day (*x*-axis) across all participants throughout the MED diet (red) and PPT diet (green) interventions. **G** As in **A** but for the average change in blood fructosamine across all participants during each of the diets (LMM, *p* < 0.001)

Comparing the effects of the two 2-week-long diets per participant, we found that all participants exhibited better responses during the PPT diet as compared to the MED diet, as reflected by one or more of the CGM-based glycemic measures (Fig. S2 and Additional file 3). For example, in 16 participants, the average PPGR across all meals was lower during the PPT intervention compared to MED (Fig. 3A). When combining data across all participants, the PPT diet leads to fewer fluctuations in glucose levels (glucose coefficient of variation, “CV”, *p* < 0.001, Fig. 3B), lower average PPGR (*p* < 0.001, Fig. 3C), lower values in PPGR percentiles (Fig. 3D), fewer hours per day with glucose levels > 140 mg/dl (Fig. 3E), and lower PPGRs throughout times of the day (Fig. 3F). Additionally, blood fructosamine levels improved significantly more during the PPT diet compared to MED, with levels decreased by 13 ± 30 μmol/dl (mean ± SD) during PPT intervention, and slightly increased by 4 ± 16 μmol/dl during MED intervention (*p* < 0.001, Fig. 3G). We performed sensitivity analyses using 4 different models to validate the results on the crossover setting. The results remained statistically significant for the treatment effect (PPT diet) on meal PPGR, glucose fluctuations, and the change in fructosamine levels (see Additional file 1: Table S2).

In terms of diet composition, the PPT diet was lower in carbohydrate content and higher in fat content on average as compared to the MED diet (22% vs. 46% of calories from carbohydrates and 51% vs. 29% of calories from fat, respectively) (Additional file 1: Table S3). These differences were also reflected in popular foods consumed in each diet (Fig. S3A, B). Notably, despite the overall lower carbohydrate content of the PPT diet, we found that meals with the same dominant food (> 70% of the meal calories) and matched for carbohydrate and energy content induced highly variable glucose responses among participants, suggesting that PPGRs are not determined exclusively by the meal carbohydrate content and that specific foods induce different PPGRs across people. For example, meals with whole-wheat bread as a dominant food, consisting of 52 ± 2.2gr of carbohydrates and 386 ± 12 kcal, induced high interpersonal variability in glucose responses across 7 participants, with a median PPGR per-person spanning a wide range, from 17 mg/dl × h to 112 mg/dl × h (Fig. S3C). Furthermore, the intra-variability (within person) was significantly lower than the inter-variability (between persons) in PPGRs to the same dominant food (mean CV, 0.27 vs. 0.46, *p* = 0.001, Fig. S3D), demonstrating personalization in glycemic responses.

### Six-month PPT intervention

Following the findings from the short-term intervention, which suggested superiority of the PPT diet over the MED diet in its effects on glucose levels, we further evaluated the long-term effects of the PPT diet on multiple metabolic parameters by an additional 6-month PPT intervention. Sixteen out of 23 participants (70%) from the short-term phase proceeded to this intervention. Notably, diet adherence during the 6-month intervention was high as assessed by self-recorded dietary intake and feedback reports for participants, with a weekly average grade of ~ 85 and calorie intake of ~ 80% from calorie target throughout the intervention (Fig. S4A, B). Interestingly, the engagement with app logging per se, evaluated by meal-related daily activities logged per participant throughout the intervention, was not correlated with improvement in glycemic parameters including daily time with glucose levels > 140 mg/dl or HbA1c (Pearson correlation, *R* = − 0.2, *p* = 0.34; *R* = 0.12, *p* = 0.65, respectively).

**Fig. 4:**
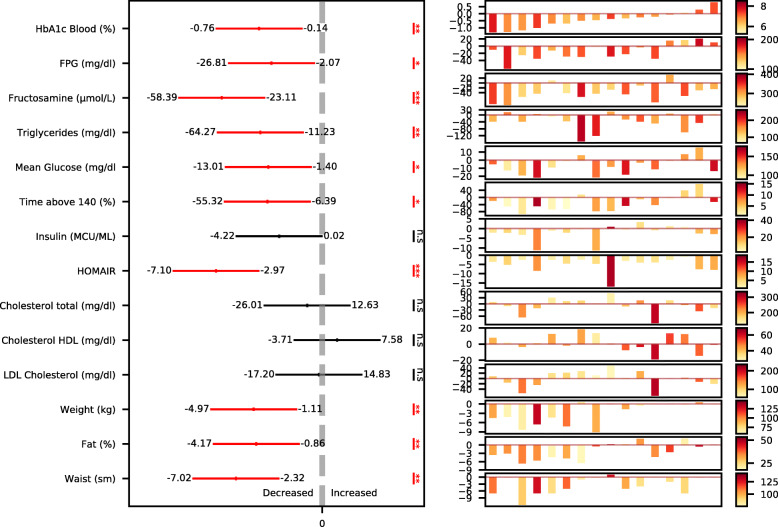
A PPT diet improves metabolic outcomes after 6 months. Illustration of changes in multiple metabolic outcomes across all participants and per participant. Left: changes in the outcomes across all participants (*n* = 16), presented as the 95% confidence interval (CI) of the change in outcomes at 6 months time point vs. baseline. Statistical significance is marked (one-sample *t*-test for all parameters except for HOMA-IR, which we used the Mann-Whitney *U* test, ***p < 0.001, **p < 0.01, *p < 0.05; n.s, not significant). Right: changes in the outcomes per participant, presented with a waterfall-like scheme, where each bar represents a participant. The color scale refers to bars, indicating the level of baseline value of each outcome for each participant. Participants are sorted by the 6-month change in HbA1c

Using blood and CGM-based measures to evaluate the effect of dietary intervention on glycemic control, we found statistically significant 6-month changes in multiple glycemic parameters, including HbA1c (mean ± SD, − 0.39 ± 0.48%, *p* < 0.001), FPG (− 16.4 ± 24.2 mg/dl, *p* = 0.02), fructosamine (− 26.7 ± 22.5 μmol/dl, *p* < 0.001), fasting insulin (− 2.3 ± 4.0MCU/ml, *p* = 0.04), HOMA-IR (− 5 ± 4.1, *p* < 0.001), mean CGM glucose (− 7.2 ± 10.9 mg/dl, *p* = 0.02), and daily time with glucose levels > 140 mg/dl (− 1.88 ± 2.89 h/day, *p* = 0.02) (Fig. 4). Importantly, 8 out of 13 (61%) participants who started the intervention with HbA1c levels ≥ 6.5% (threshold for T2DM diagnosis) reached HbA1c levels < 6.5% at the end of the intervention, indicating diabetes remission. Additionally, in the serum lipid profile, we found a significant reduction in triglycerides (− 49 ± 46 mg/dl, *p* < 0.001), with no significant changes in HDL and total cholesterol. Notably, despite high levels of dietary fat (> 50% of energy), including saturated fat (> 10% of energy), consumed by participants during the PPT intervention, there was no significant 6-month change in LDL cholesterol, with the mean change trending for reduction (− 4.7 mg/dl, *p* = 0.4, Fig. 4). Other metabolic readouts that showed significant 6-month reductions were body composition measurements, including body weight (− 3 ± 3.5 kg, *p* = 0.005), body fat % (− 2.5 ± 3%, *p* = 0.005), and waist circumference (− 4.7 ± 3.7 cm, *p* = 0.001) (Fig. 4). Notably, 50% of the participants (8 out of 16) lost weight (> 1 kg) during the intervention, but 4 out of 8 who did not lose weight did exhibit reductions in HbA1c (> 0.2%).

To assess whether the improvements in glycemic measures were indeed driven by the PPT diet approach rather than a simple Hawthorne effect, we compared between participants with higher vs. lower rates of dietary adherence. Although dietary adherence was relatively high among all participants and despite having a small cohort, we were able to detect significant differences in glycemic parameters changes between participants with higher vs. lower dietary adherence, determined by the average feedback grade above or below the group median (= 84.4). This included daily time with glucose levels > 140 mg/dl (mean ± SD, − 3.6 ± 2.7 h/day vs. − .4 ± 2.6 h/day, respectively; *p* = 0.028), FPG (− 25.3 ± 19 mg/dl vs. − 4.8 ± 26.5 mg/dl, respectively; *p* = 0.036), fasting insulin (− 3.9 ± 4 vs. − 0.2 ± 3.3, respectively; *p* = 0.046) and HOMA-IR (− 2.3 ± 0.2 vs. 2.3 ± 0.2 mg/dl, respectively; *p* = 0.02). There was also a trend for a greater reduction in HbA1c levels in the higher vs. lower adherence group, but these did not reach statistical significance (− 0.6 ± 0.48 vs. − 0.18 ± 0.42, respectively; *p* = 0.059) (Fig. S4C). Notably, weight change did not differ significantly between these adherence groups (− 3.6 ± 4.1 kg vs. − 2.3 ± 2.4 kg, respectively; *p* = 0.2).

### Gut microbiome changes

**Fig. 5:**
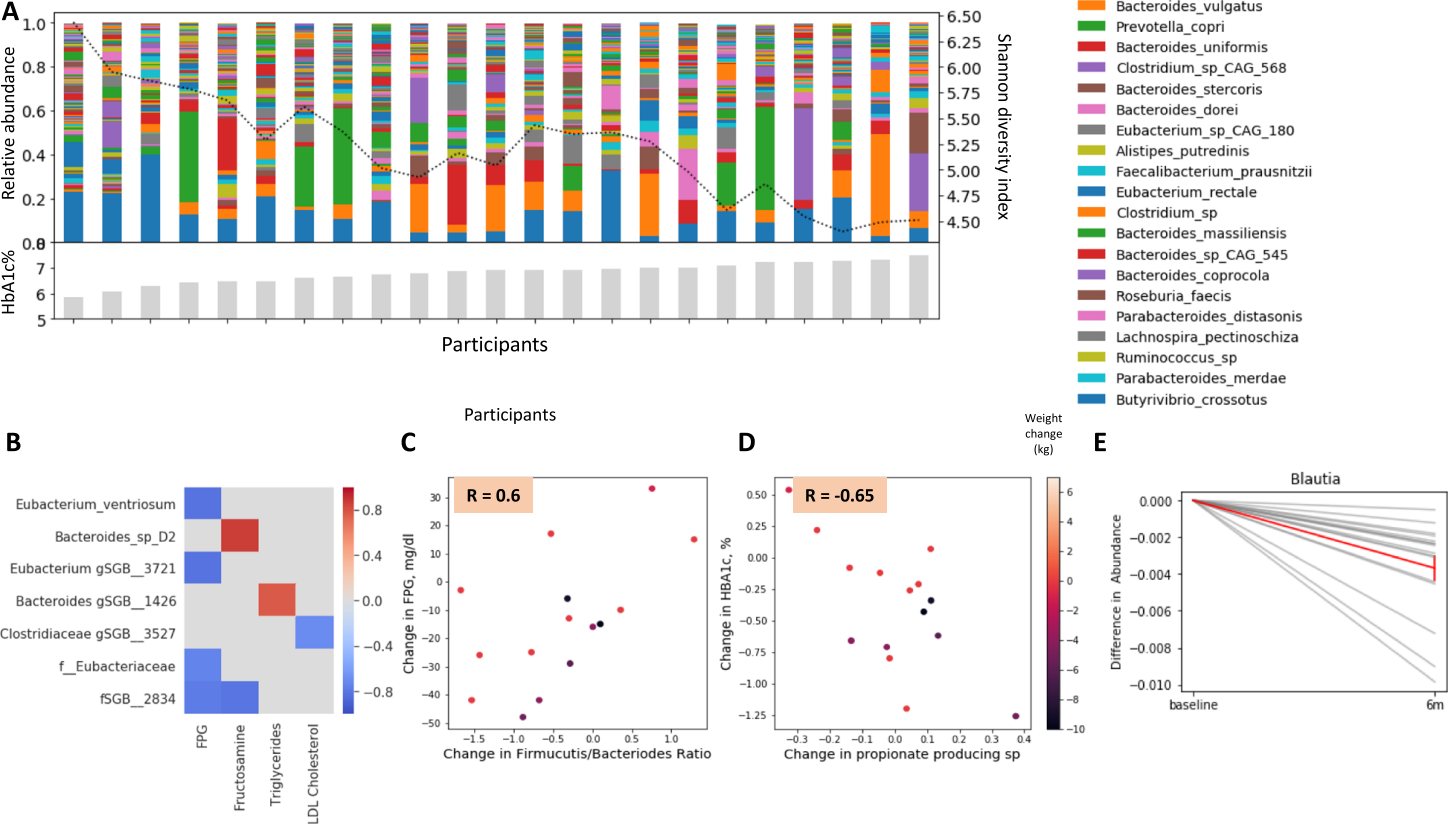
Gut microbiome composition associates with clinical outcomes. **A** Per participant distributions of microbial taxa relative abundances at baseline. Colors indicate bacterial taxa according to the legend on the right. Participants are sorted by baseline HbA1c levels, presented as gray bars at the bottom. Microbiome diversity (Shannon Diversity Index) is illustrated with a dashed line, using a 5-person rolling average, indicating a negative association with HbA1c levels. **B** Heatmap of significant associations across all (*n* = 16) participants (*p* < 0.05, FDR corrected) between changes in microbial taxa (rows) and changes in clinical outcomes (columns) over the 6-month intervention period. **C** Correlation between 6-month change in FPG and 6-month change in Firmicutes/Bacteroidetes ratio. Dots represent participants, with color indicating weight loss in kilograms. **D** Correlation between change in HbA1c and change in propionate-producing bacteria over the 6-month intervention period. Dots represent participants, with color indicating weight loss in kilograms. **E** Change in the relative abundance of *Blautia* between baseline and 6 months after the intervention started (*p* < 0.05, FDR corrected). Shown is the average reduction in the relative abundance of *Blautia* genus across all participants (red line) and change per participant (gray lines)

We sought to evaluate the effects of the PPT intervention on gut microbiome composition and its link to clinical changes. First, we performed a relative abundance analysis to evaluate the bacterial composition at the baseline of all 23 participants. We found that microbiota diversity was negatively associated with HbA1c levels (Fig. 5A), consistent with other studies demonstrating associations between low microbiome diversity and poor glycemic control, inflammation, and adiposity [33]. Next, we used the microbiome samples collected at 6 months (*n* = 16) to evaluate whether the intervention induced significant changes to microbiome composition, and whether these changes were associated with clinical outcomes. We detected several significant associations between changes in microbial taxa and changes in clinical outcomes, across all participants (*p* < 0.05, FDR corrected at 0.15, Fig. 5B). For example, changes in *Eubacterium ventriosum* were negatively associated with changes in FPG levels. Notably, this finding is in agreement with another study which suggested that lower levels of this species at baseline were predictive of improvement in insulin sensitivity after fecal microbiota transplantation (FMT) in patients with metabolic syndrome [34]. We also found a significant positive correlation (*R* = 0.60, *p* = 0.02) between 6-month changes in FPG and changes in Firmicutes/Bacteroidetes ratio, the latter commonly associated with poor metabolic health, including low-grade inflammation and obesity [35, 36]. A negative significant correlation (*R* = − 0.65, *p* = 0.0008) was found between 6-month changes in HbA1c and changes in propionate-producing bacteria, the latter considered to have health-promoting functions [37]. Both of these associations were independent of weight loss in this cohort (Fig. 5C, D). Additionally, while many of the changes in microbiome composition were person-specific, several microbial taxa had the same direction of change across all participants. For example, the relative abundance of *Blautia*, one of the most abundant genera in the gut, decreased across all participants (Fig. 5E)*.* Notably, it was shown to be positively associated with T2DM [38].

## Discussion

In this short-term crossover dietary intervention in adults with newly diagnosed T2DM and naive to glucose-lowering medications, an algorithm-based personalized postprandial targeting (PPT) diet, successfully improved CGM-based measures of average PPGR, glucose fluctuations, and daily time with glucose levels > 140 mg/dl, as compared to a commonly recommended Mediterranean-style (MED) diet. Blood fructosamine levels also improved significantly more during the PPT diet compared to the MED diet. Additional 6-month PPT intervention resulted in significant improvements in multiple metabolic parameters, including HbA1c, fasting glucose, HOMA-IR, daily time with glucose levels > 140 mg/dl, and blood triglycerides. Notably, out of 16 participants in the long-term intervention, 13 had HbA1c levels > 6.5% (threshold for diabetes diagnosis) at baseline, and 8 of them (61%) reached HbA1c levels < 6.5% at 6 months, indicating diabetes remission. Interestingly, despite no calorie restriction applied, half of the participants (8 out of 16) lost weight (> 1 kg) during the 6-month intervention, and 9 participants (56%) reduced body fat % (> 2.5%) or waist circumference (≥ 2 cm). Notably, 4 out of 8 participants who did not lose weight did exhibit reductions in HbA1c (> 0.2%) at 6 months. Lastly, improvements in clinical outcomes were accompanied by microbiome alterations, some of which are in line with previous publications. For example, reductions in Firmicutes/Bacteroidetes ratio [35, 36] and increases in propionate-producing bacteria [37] previously reported to be health-promoting effects. Importantly, comparisons were made within participants, which gives a good reference since changes in microbiome composition per person are typically expected to be small [39].

Our findings support the fundamental role of dietary modifications for improving glycemic control in newly diagnosed T2DM and possibly leading to diabetes remission or delaying need of diabetes medications, as previously demonstrated by several studies [7, 8, 40]. In a large-scale dietary intervention in adults with newly diagnosed T2DM, Andrews et al. reported a 6-month mean change of − 0.28% and − 0.33% in HbA1c following an intensive diet or intensive dietary plus physical activity interventions [40], as compared with a 6-month mean change of − 0.39% reported in here. Importantly, the interventions by Andrews et al. consisted of both calorie restriction and usual care with diabetes medications, as opposed to the current study, suggesting that non-calorie-restricted interventions directly targeting reductions in PPGRs may be more effective than standard medical and nutritional care for improving glycemic control in newly diagnosed T2DM. In terms of diabetes remission, Taheri et al. reported in a clinical trial in individuals with short-duration diabetes (≤ 3 years) that diabetes remission occurred in 61% of participants following a 1-year intensive lifestyle intervention consisting of total diet replacement phase with low-energy formulas and a weight-loss maintenance phase involving food reintroduction combined with physical activity [8]. In the present study, although of a much lower scale, we report comparable rates of diabetes remission after 6 months (61%) with a much more tolerable dietary approach. In another study, Esposito et al. reported in a dietary intervention in individuals with T2DM and naive to diabetes medications, much lower rates of diabetes remission after 1-year intervention with a low-carbohydrate Mediterranean diet (14.7%) or a low-fat diet (4.1%) [7].

Our study has several strengths. First, the use of CGM throughout the interventions allowed to directly evaluate the “real-time” glucose responses to many meals and assess the effect of reductions in PPGRs on other metabolic parameters in time courses of weeks and months. In addition, the study design included full-time self-recorded dietary intake using a smartphone application, which allowed to track dietary adherence precisely. This provided much more accurate assessment of the actual dietary consumption throughout the interventions, as compared to other dietary intake assessment tools that are typically used in nutritional studies, such as food frequency questionnaires (FFQ) or occasional 24-h recalls.

Our study also has several limitations. As designed to be a pilot study as a “proof-of-concept” for the clinical efficacy of the algorithm-based diet in newly diagnosed T2DM, it is a small-scale study including 23 participants in the short-term intervention. Nevertheless, the crossover design provided sufficient statistical power to capture the differences between the two dietary strategies even in this small cohort. Also, the long-term intervention program did not include a control group to compare the long-term effects of the PPT diet to those of a MED diet, since blinding was removed and results revealed to participants at the end of the crossover intervention. This restricted us to proceed to the long-term intervention with a single-arm design, to further explore the clinical long-term effects of the PPT diet. Lastly, as described, the PPT diet does not rely on definition on predetermined macronutrient distributions, and since meal carbohydrate content constitutes an important (but not exclusive) factor in PPGR prediction, the PPT diet resulted in a relatively lower carbohydrate content (22% of energy) as compared to the MED diet (46% of energy). It is thus possible that the beneficial effects observed with the PPT diet are mainly driven by their lower carbohydrate content. However, we speculate that this is not the case, since meals with the same dominant food and matched for energy and carbohydrate content induced highly variable PPGRs between participants (Fig. S3C, D). Nevertheless, larger-scale extended studies are needed to validate the clinical efficacy of the PPT diet approach as compared to other dietary strategies and for estimation of its effect on microbiome composition.

## Conclusions

In this crossover dietary intervention in newly diagnosed T2DM subjects, a PPT diet improved glycemic measures significantly more than a Mediterranean diet and further improved various metabolic parameters in an additional 6-month intervention. These findings may be valuable for the design of future larger studies that may have implications for dietary advice in clinical practice.

## Supplementary Information

**Additional file 1: Fig. S1.** Menus construction and scoring. **Fig. S2.** PPT diet improves glucose excursions in newly diagnosed T2DM subjects. **Fig. S3.** Dominant food. **Fig. S4.** Monitoring Diet Adherence. **Table S1.** Characteristics of participants who did not proceed to the 6-month intervention. **Table S2.** Sensitivity analysis for the crossover outcomes. **Table S3.** Dietary intake during crossover intervention.

**Additional file 2.** List of features used for predictions of personal postprandial response.

**Additional file 3.** Data of glucose features in crossover intervention.

## Data Availability

The datasets generated during and/or analyzed during the current study are available from the corresponding upon request.

## Abbreviations

ALT: Alanine aminotransferase
AST: Aspartate aminotransferase
BMI: Body mass index
CGM: Continuous-glucose-monitoring
CRP: C-reactive protein
CT: Caloric target
CV: Coefficient of variation
FDR: False discovery rate
FFQ: Food frequency questionnaires
FMT: Fecal microbiota transplantation
FPG: Fasting plasma glucose
GGT: Gamma-glutamyl transferase
HDL-cholesterol: High-density lipoprotein cholesterol
HOMA-IR: Homeostatic model assessment for insulin resistance
IRB: Institutional review board
LDL cholesterol: Low-density lipoprotein cholesterol
MED diet: Mediterranean diet
PNP: Personalized nutrition project
PPGR: Postprandial glucose responses
PPT diet: Personalized postprandial-targeting diet
RA: Relative abundance
T2DM: Type 2 diabetes mellitus
