# Supplementary material for: Effects of personalized diets by prediction of glycemic responses on glycemic control and metabolic health in newly diagnosed T2DM: a randomized dietary intervention pilot trial
Source: BMC Med. 2022 Feb 9;20:56. doi: 10.1186/s12916-022-02254-y (PMC8826661; doi:10.1186/s12916-022-02254-y)
Supplement: Supplementary file 1 — Additional file 1: Fig. S1. Menus construction and scoring. Fig. S2. PPT diet improves glucose excursions in newly diagnosed T2DM subjects. Fig. S3. Dominant food. Fig. S4. Monitoring Diet Adherence. Table S1. Characteristics of participants who did not proceed to the 6-month intervention. Table S2. Sensitivity analysis for the crossover outcomes. Table S3. Dietary intake during crossover intervention. [file 12916_2022_2254_MOESM1_ESM.docx]

**Additional file 1**

**Effects of personalized diets by prediction of glycemic responses on glycemic control and metabolic health in newly-diagnosed T2DM: a dietary intervention pilot trial**

*Correspondence should be addressed to:

Prof. Eran Elinav, [eran.elinav@weizmann.ac.il](mailto:eran.elinav@weizmann.ac.il) or Prof. Eran Segal, [eran.segal@weizmann.ac.il](mailto:Eran.Segal@weizmann.ac.il)

**Table of content**

**Supplemental Methods**

Complete list for study eligibility 3

Menus construction 3

**Supplementary Figure 1**. Menus construction and scoring 5

**Supplementary Figure 2**. PPT diet improves glucose excursions in newly diagnosed T2DM subjects. 6

**Supplementary Figure 3**. Dominant foods 7

**Supplementary Figure 4**. Monitoring Diet Adherence 8

**Supplementary Table 1**. Characteristics of participants who did not proceed to the 6-month intervention 9

**Supplementary Table 2**. Sensitivity analysis for the crossover outcomes 10

**Supplementary Table 3**. Dietary intake during crossover intervention 11

**Supplemental Methods**

**Complete list for study eligibility**

All study participants met the following inclusion criteria: age 18-65; HbA1c, 6.5-8% or FPG, 140-180 mg/dl; naive to glucose-lowering medications; and capability to work with a smartphone application on a daily basis (for food logging). Exclusion criteria included: **(1)** Recent treatment (last 3 months) with antibiotics/antifungal; **(2)** Use of anti-diabetic and/or weight-loss medication; **(3)** Proteinuria (at screening); (**4)** Chronic use of medications that affect glucose metabolism (e.g. steroids, thiazide diuretics). Other drugs that may have some effect on glucose metabolism but are very commonly used and therefore were not excluded from the study: oral contraceptive, antidepressants (e.g. SSRI's), methylphenidate (Ritalin), cardiovascular medications: beta-blockers, statins, and vasodilators); **(5)** People under another diet regime and/or a dietitian consultation/another study; **(6)** Pregnancy or fertility treatments; **(7)** Chronic diseases/medical conditions that affect energy/glucose metabolism or that require specific and intensive dietary management (HIV, Cushing syndrome, CKD, acromegaly, hyperthyroidism or imbalanced hypothyroidism, liver cirrhosis, inflammatory bowel diseases, cardiomyopathy, cardiac arrhythmia, COPD, Triglycerides>500 mg/dl). Other medical conditions (comorbidities) which are highly prevalent (e.g. dyslipidemia, hypertension, etc.) or that require minor dietary adaptations (e.g. nephrolithiasis), were not excluded. In these cases, the dietitian provided participants with additional dietary advice on how to adapt the assigned diet and comply with specific dietary requirements for these conditions; **(8)** Anemia (Hb<11.7 for women, Hb<13.1 for men); **(9)** Hemoglobinopathies (including sickle cell anemia and thalassemia); **(10)** Cancer and recent anticancer treatment in the last 5 years; **(11)** Psychiatric disorders (including antipsychotic medication such as- chlorpromazine, perphenazine, clozapine, iloperidone, olanzapine, paliperidone, quetiapine, risperidone); **(12)** Coagulation disorders; **(13)** Bariatric surgery; **(14)** Alcohol or substance abuse.

**Menus construction**

**Meal bank (list)**

The menus provided to participants in this study were constructed from a meal bank that we generated, with over 2,000 meals representative of the Israeli typical diet and with a variety of different food combinations. We divided the meals in the meal bank into 4 meal types (breakfast, lunch, dinner and snacks) and labeled them according to meal categories (dairy; meat; fish etc.) which enabled us to generate menus according to participants' personal preferences. All meals in the meal bank were scored for each participant, using a score scale of 1 to 5 (best to worst) based on the scoring principles of the MED diet and PPT diet as described hereafter. Menus included only meals with scores 1 and 2.

**Scoring system for the PPT diet**

The scoring system on the PPT diet is size-independent aimed to avoid the effect of meal calorie content on prediction and compare between meals in an unbiased manner. All meals are theoretically scaled for each participant separately in the meal bank to fixed calorie contents (20%, 25% and 30% of daily caloric target for main meals; 10% and 15% of daily caloric target for snacks) and computed the predicted glycemic response for each meal in all caloric targets. We then used the distribution of meals by their average predicted response (separate distributions for main meals and snacks) and divided them into 5 percentiles of 10%, 15%, 25%, 25%, 25% to assign meal scores of 1 to 5 (best to worst), respectively.

**Algorithm for PPGR predictions**

Algorithm for personalized prediction of postprandial glucose response (PPGR) originated from a previous work in our lab and was adjusted for the usage in a clinical setting. Among the features used to predict PPGR to meals were anthropometrics, blood tests (FPG, HbA1c% and Hemoglobin), lifestyle features derived from questionnaires, microbiome (abundances of species estimated by MetaPhlAn2 and meal features (macro- and micronutrient composition) were used. Since no events around the meal were used for prediction, trained predictor could predict response for any profiled participant to any given meal.

We used stochastic gradient boosting regression, such that 80% of the samples and 40% of the features were randomly sampled for each estimator. The depth of the tree at each estimator was not limited, but leaves were restricted to have at least 60 instances (meals). We used 5000 estimators with a learning rate of 0.005. Xgboost library v 0.6 was used.

**Caloric target calculation**

The caloric target for each participant was computed as the mean of:

**(a)** Mifflin equation for Estimating Energy Requirements (EER). A predictive equation for Resting Energy Expenditure (REE) for healthy individuals, using their weight, height, age and gender, and multiplied by Physical Activity (PA) factor based on the level of PA that the person does on a regular basis.

**(b)** Energy expenditure assessed by Basal Metabolic Rate (BMR) value measured by body composition analyzer (Tanita), divided by 0.7 since BMR accounts for 65-70% of total energy expenditure.

**(c)** The regular daily caloric intake obtained from 3 days of dietary records in the logging app during the profiling stage, to account for the person’s dietary habits prior to the intervention.

**Figure S1. PPT menu construction and scoring**


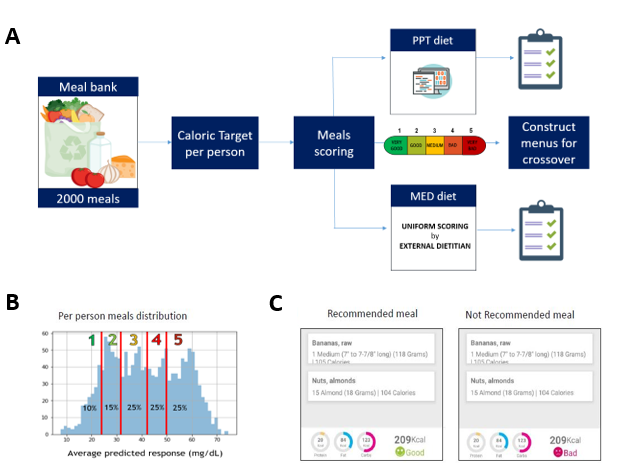


**Figure S1. PPT menu construction and scoring.** **(A)** The menus construction flow **(B)** An example of different outputs (scores) in the ‘traffic light’ feature on the smartphone application for one identical meal for 2 different participants. **(C)** Illustration of the meal scoring system for the PPT diet. Presented is an example of a per person distribution of the predicted PPGRs to meals from the meal bank, which served for determining meal scores on a scale of 1 to 5.

**Figure S2. PPT diet improves glucose excursions in newly diagnosed T2DM subjects.**


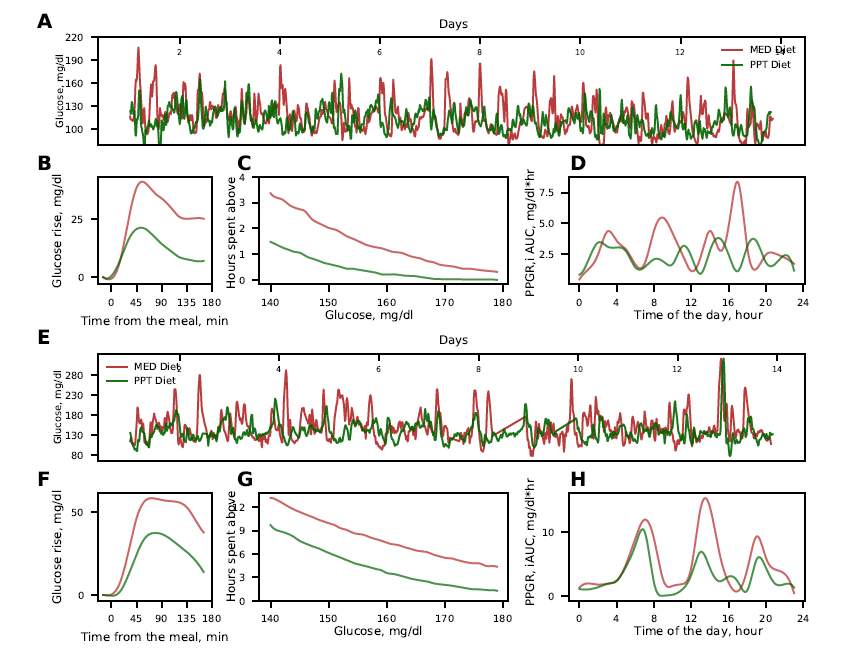


**Figure S2. PPT diet improves glucose excursions in newly diagnosed T2DM subjects.** Comparison of CGM-based glucose measures during PPT diet vs MED diet, for two selected participants, one from the ‘PPT-MED’ group (A-D) and one from the ‘MED-PPT’ group (E-H). **(A)** Continuous glucose measurements of one participant from the ‘PPT-MED’ group across the entire two weeks of MED diet (red) and PPT diet (green). **(B)** Average glucose rise after meals for the same participant as in A during the MED diet (red) and PPT diet (green). **(C)** Number of daily hours (y axis) above glucose level thresholds (x axis) for the same participant as in A during the MED diet (red) and PPT diet (green). **(D)** Average PPGR (y axis) during hours of the day (x axis) for the same participant as in A during the MED diet (red) and PPT diet (green). **(E-H)** Same as A-D, respectively, but for one participant from the ‘MED-PPT’ group.

**Figure S3. Dominant foods.**

**
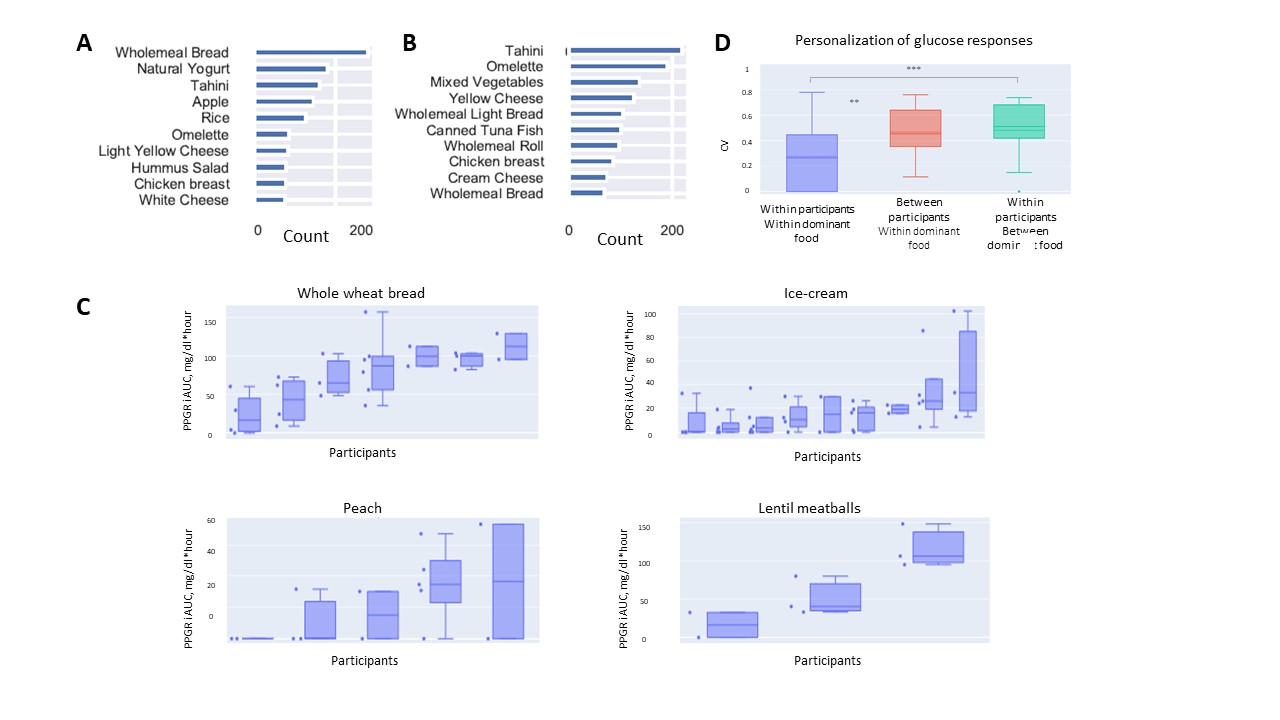
**

**Figure S3. Dominant foods.** **(A)** Top 10 popular foods on the MED diet menus during the crossover intervention. **(B)** Top 10 popular foods on the PPT diet menus during the crossover intervention. **(C)** Box plots by individuals, indicating the variability between people in glycemic responses to meals with a dominant food (>70% of meal calorie content) and matched for meal calories and dietary carbohydrate content. **(D)** Box plots by groups of participants/foods indicating the intra and inter variability (CV) in PPGRs to dominant foods.

**Figure S**4**. Monitoring Diet Adherence**


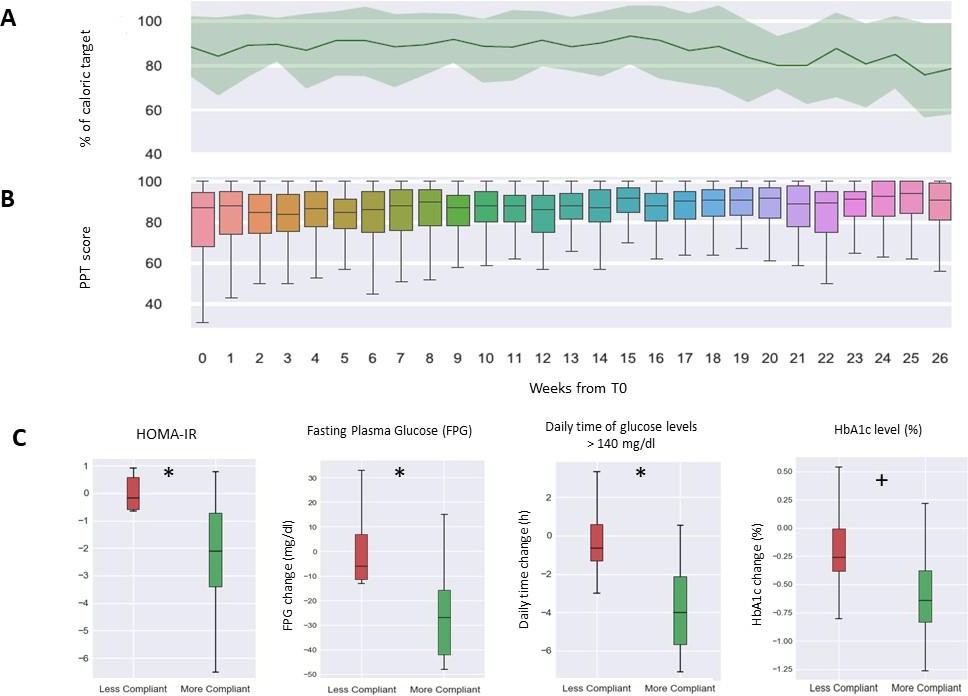


**Figure S**4**. Monitoring Diet Adherence. (A)** Mean amount of calories logged by participants in the 6-month intervention, as percent of caloric target. **(B)** Box plots of the mean PPT diet grades across all participants throughout the 6-month intervention period (26 weeks). Vertical bars indicate standard errors. **(C)** Box plots of changes in glycemic markers in participants with a higher adherence to the dietary recommendations (green; n=8) vs participants with a lower adherence to the dietary recommendations (red; n=7). One participant had been excluded from this analysis only due to an HbA1c levels below 6.5% (=5.78). Statistical significance is marked (Mann Whitney U-test *p<0.05, + p<0.1).

**Table S1: Baseline characteristics and glycemic markers of participants who proceeded or did not proceed to the additional 6-month intervention**

| **Parameters (units)** | **Proceeded to Long Term (n=16)** | **Not Proceeded (n=7)** | **P-value*** |
| --- | --- | --- | --- |
| Age (y) | 53.8±7.7 | 52.2±13.6 | 0.72 |
| Male sex number (%) | 6 (44) | 4 (60) | 0.55 |
| **Anthropometric measurements** | | | |
| Weight (kg) | 89.3±21.6 | 81±22 | 0.45 |
| BMI (kg/m2) | 31.1±7.4 | 29.6±8.1 | 0.69 |
| Waist circumference (cm) | 103.3±13.6 | 96.8±20.5 | 0.43 |
| Body Fat (%) | 34.3±10.4 | 31.6±13.8 | 0.63 |
| **Lipid profile** | | | |
| Serum total cholesterol (mg/dl) | 197.2±38.7 | 221±43.5 | 0.24 |
| Serum LDL cholesterol (mg/dl) | 122.2±30.7 | 132±29.9 | 0.53 |
| Serum HDL cholesterol (mg/dl) | 46.2±8.6 | 52.5±9.3 | 0.16 |
| Serum triglycerides (mg/dl) | 166.1±57.1 | 163±90.5 | 0.92 |
| **Glucose biomarkers** | | | |
| HbA1c (%) | 6.8±0.4 | 6.6±0.4 | 0.3 |
| Fructosamine (µmol/L) | 301.6± 44.1 | 297.2±27.3 | 0.09 |
| Fasting glucose (mg/dl) | 151.4±19.9 | 143.4±15.7 | 0.41 |
| **Glycemic markers** | | | |
| PPGR iAUC (mg/dl*hour) | -29±18 | -18±16 | 0.17 |
| Daily time of glucose levels above 140mg/dl (hour /day) | 2.6±2.6 | 2±1.8 | 0.27 |
| Fructosamine change (µmol/L) | -35±63 | -12±30 | 0.27 |

*P-values for differences between groups were calculated using t-test for baseline characteristic and Mann-Withney for CGM-based parameters.

Glycemic parameters present as the calculated change during each diet (PPT or MED), as measured by CGM.

Abbreviations: BMI - body mass index, PPT- personalized postprandial-targeting diet, MED - Mediterranean diet, CGM – continues glucose monitoring device.

**Table S2: Sensitivity analysis** **for crossover outcomes**

| **Crossover outcomes** | **N** | **Statistical method** | **95% confidence interval** | | **P-value** |
| --- | --- | --- | --- | --- | --- |
| Fructosamine change (µmol/L) | 23 | Paired t test | -32.41 | -0.11 | 0.048 |
|  |  | LMM (baseline values) | -42.5 | -11.9 | <0.001 |
|  |  | LMM (baseline, Age, sex) | -43.9 | -11.7 | 0.001 |
|  |  | Wilcoxon Signed-Rank Test | N.A | N.A | <0.001 |
| PPGR iAUC (mg/dl*hour) | 23 | Paired t test | -0.04 | -0.01 | <0.001 |
|  |  | LMM | -0.08 | -0.024 | <0.001 |
|  |  | LMM (age, sex) | -0.067 | -0.018 | 0.001 |
|  |  | Wilcoxon Signed-Rank Test | N.A | N.A | <0.001 |
| Blood glucose fluctuations (coefficient of variation) | 23 | Paired t test | -26.86 | -12.79 | <0.001 |
|  |  | LMM | -39.312 | -14.941 | <0.001 |
|  |  | LMM (age, sex) | -37.992 | -13.183 | <0.001 |
|  |  | Wilcoxon Signed-Rank Test | N.A | N.A | <0.001 |

**Table S2. Sensitivity analysis for crossover outcomes** including different statistical models.

PPT- personalized postprandial-targeting diet, MED – Mediterranean

Linear mixed model (LMM) included the period (first or second intervention), dietary approach-treatment (PPT or MED) and the sequence of the treatment (PPT-MED or MED-PPT). Covariates (if included) were baseline value, age and sex.

**Table S3. Dietary intake during crossover intervention**

|  | **PPT –diet**, Mean(SD) | **MED-diet**, Mean(SD) | **P-value*** |
| --- | --- | --- | --- |
| **Total Energy intake** | | | |
| kcal/day | 2,105.7±332.45 | 1,979.87±414.59 | 0.27 |
| **Carbohydrate** | | | |
| g/day | 115.31±20.02 | 227.83±46.74 | <0.001 |
| % of energy | 21.90% | 46.03% | <0.001 |
| **Protein** | | | |
| g/day | 129.04±38.56 | 99.84±22.11 | 0.003 |
| % of energy | 24.51% | 20.17% | <0.001 |
| **Total fat** | | | |
| g/day | 119.72±16.95 | 65.61±18.70 | <0.001 |
| % of energy | 51.17% | 29.83% | <0.001 |
| **Saturated Fat** |  |  |  |
| g/day | 33.96±6.14 | 15.12±3.90 | <0.001 |
| % of energy | 14.52% | 6.87% | <0.001 |
| **Fiber** | | | |
| g/day | 18.94±5.99 | 37.73±11.63 | <0.001 |

*P-values for differences between diets were calculated using t-test. PPT- personalized postprandial-targeting diet, MED – Mediterranean
