# Supplementary material for: Effects of personalized diets by prediction of glycemic responses on glycemic control and metabolic health in newly diagnosed T2DM: a randomized dietary intervention pilot trial
Source: BMC Med. 2022 Feb 9;20:56. doi: 10.1186/s12916-022-02254-y (PMC8826661; doi:10.1186/s12916-022-02254-y)
Supplement: Supplementary file 3 — Additional file 3. Data of glucose features in crossover intervention. [file 12916_2022_2254_MOESM3_ESM.docx]

**Additional file 3. Data of glucose features in crossover intervention**

| **Subject no.** | **Glucose fluctuation (CV) PPT diet** | **Glucose fluctuation (CV) MED diet** | **Glucose fluctuation (CV) Δ PPT diet vs. MED diet** | **Meal PPGR PPT diet** | **Meal PPGR MED diet** | **Meal PPGR Δ PPT diet vs. MED diet** | **Daily time of glucose >140 mg/dl PPT diet** | **Daily time of glucose>140 mg/dl MED diet** | **Daily time of glucose>140 mg/dl Δ PPT diet vs. MED diet** | **Mean glucose PPT diet** | **Mean glucose MED diet** | **Mean glucose Δ PPT diet vs. MED diet** |
| --- | --- | --- | --- | --- | --- | --- | --- | --- | --- | --- | --- | --- |
| Units | sd/mean | sd/mean |  | mg/dl*hour | mg/dl*hour |  | hour/day | hour/day |  | mg/dl | mg/dl | mg/dl |
| 1 | 0.19 | 0.2 | -0.01 | 40.99 | 40.63 | 0.36 | 10.41 | 10.64 | -0.23 | 131.18 | 133.71 | -2.53 |
| 2 | 0.17 | 0.25 | -0.08 | 31.45 | 67.81 | -36.36 | 9.7 | 13.21 | -3.51 | 123.3 | 134.97 | -11.67 |
| 3 | 0.24 | 0.26 | -0.02 | 46.86 | 80.28 | -33.42 | 7.93 | 7.3 | 0.63 | 139.68 | 139.08 | 0.6 |
| 4 | 0.24 | 0.24 | 0 | 43.13 | 79.44 | -36.31 | 9.04 | 12.35 | -3.31 | 144.75 | 157.4 | -12.65 |
| 5 | 0.16 | 0.22 | -0.06 | 38.06 | 69.33 | -31.27 | 11.12 | 15.76 | -4.64 | 143.3 | 153.66 | -10.36 |
| 6 | 0.18 | 0.17 | 0.01 | 39.99 | 40.37 | -0.38 | 21.82 | 21.92 | -0.1 | 146.07 | 144.4 | 1.67 |
| 7 | 0.2 | 0.24 | -0.04 | 52.18 | 57.64 | -5.46 | 8.03 | 9.89 | -1.86 | 124.91 | 133.34 | -8.43 |
| 8 | 0.17 | 0.21 | -0.04 | 31.83 | 55.34 | -23.51 | 2.41 | 5.72 | -3.31 | 106.63 | 114.27 | -7.64 |
| 9 | 0.2 | 0.27 | -0.07 | 46.21 | 57.25 | -11.04 | 7.38 | 11.25 | -3.87 | 129.39 | 144.6 | -15.21 |
| 10 | 0.22 | 0.23 | -0.01 | 59.84 | 93.26 | -33.42 | 6.81 | 8.67 | -1.86 | 130.56 | 132.97 | -2.41 |
| 11 | 0.27 | 0.22 | 0.05 | 43.06 | 36.32 | 6.74 | 11.61 | 13.11 | -1.5 | 146.33 | 146.17 | 0.16 |
| 12 | 0.16 | 0.24 | -0.08 | 27.02 | 51.89 | -24.87 | 5.94 | 13.23 | -7.29 | 122.69 | 143.38 | -20.69 |
| 13 | 0.14 | 0.19 | -0.05 | 21.99 | 46.55 | -24.56 | 1.48 | 3.36 | -1.88 | 112.62 | 117.25 | -4.63 |
| 14 | 0.22 | 0.26 | -0.04 | 28.14 | 63.55 | -35.41 | 1.14 | 1.95 | -0.81 | 84.22 | 86.66 | -2.44 |
| 15 | 0.19 | 0.24 | -0.05 | 22.49 | 65.6 | -43.11 | 3.31 | 6.14 | -2.83 | 119.81 | 126.24 | -6.43 |
| 16 | 0.21 | 0.24 | -0.03 | 54.36 | 71.87 | -17.51 | 6.91 | 8.81 | -1.9 | 136.15 | 141.84 | -5.69 |
| 17 | 0.2 | 0.2 | 0 | 40.07 | 55.69 | -15.62 | 1.57 | 1.79 | -0.22 | 98.41 | 99.32 | -0.91 |
| 18 | 0.29 | 0.34 | -0.05 | 42.91 | 93.75 | -50.84 | 2.98 | 7.09 | -4.11 | 108.92 | 125.99 | -17.07 |
| 19 | 0.2 | 0.18 | 0.02 | 41.44 | 32.25 | 9.19 | 0.37 | 1.05 | -0.68 | 108.04 | 108.6 | -0.56 |
| 20 | 0.19 | 0.24 | -0.05 | 25.61 | 44.4 | -18.79 | 3.56 | 6.05 | -2.49 | 117.49 | 123.32 | -5.83 |
| 21 | 0.18 | 0.22 | -0.04 | 47.72 | 54.38 | -6.66 | 3.55 | 6.07 | -2.52 | 106.78 | 114.36 | -7.58 |
| 22 | 0.19 | 0.17 | 0.02 | 15.63 | 23.69 | -8.06 | 3.92 | 3.74 | 0.18 | 119.68 | 119.38 | 0.3 |
| 23 | 0.2 | 0.22 | -0.02 | 36.8 | 52.47 | -15.67 | 2.99 | 4.88 | -1.89 | 99.11 | 105.53 | -6.42 |

PPT- personalized postprandial-targeting diet, MED – Mediterranean. PPGR – postprandial glucose response. Green background represents better levels during the PPT diet connection; Red background represents better levels during the MED diet connection.
